# Rectal Feeding

**Published:** 1901-11-02

**Authors:** 


### Hospital Clinics and Medical Progress.

### RECTAL FEEDING.

So far as careful adaptation of means to an end is concerned, few prescriptions are apt to be so thoughtlessly given as those for feeding by the bowel. Even the standard hospital formulæ are in some cases far from being such as would be suggested by a scientific consideration of the problem which has to be solved, and it is quite clear that if in such cases the whole benefit which this form of alimentation can provide is to be obtained by the patient, much more serious attention must be given to the details of the process than they usually receive. Dr. A. P. Beddard[1] says that at the best a diet wholly given by the rectum is but little removed from starvation, and that as the rectum is only able to absorb in a day an amount of food containing about 500 to 800 kilo-calories of energy, which is not more than a third of what an adult lying at rest in bed requires, the greatest care must be exercised that the injections shall consist of materials capable of being absorbed and that they shall be administered in such a manner as not to set up actions which might interfere with their utility.

Proteids vary greatly in the readiness with which they are absorbed by the large intestine. All peptonised proteids are readily absorbed, as are raw meat juice and beaten up eggs. On the other hand, gelatin is not absorbed at all, and the proteids of undigested milk most imperfectly. Of dry peptone powders Dr. Beddard mentions somatose made from meat, milk somatose, and Mosquera beef-meal as being useful. The last-named, however, contains a good deal of fat, and fat being very slowly absorbed by the rectum is of but small advantage in enemata. Of artificial beef-juices the only one that contains a large percentage of true proteid is Puro. In regard to eggs the yolk differs from the rest only in its far higher percentage of fat, and thus offers no advantage for rectal feeding.

Among carbohydrates cane sugar is inadmissible because, although readily absorbed, it is taken up without alteration and is not assimilable. Dextrose is assimilable and rapidly absorbed, but it has a high osmotic pressure, so that it can only be used in such weak solution as not to be of much value as a food. If stronger solutions are employed they will for a time attract large quantities of water into the bowel and the enema may soon be returned. Starch, curiously enough, is well absorbed when given raw. Being colloid it has no omotic pressure, and thus can be given in large quantities at a time.

Fats, from their high calorific value, would be of great help in rectal feeding were it not that the rectal mucous membrane absorbs them so slowly. Under the most favourable circumstances the rectum cannot absorb more than about ½ oz. of fat in 24 hours, *i.e.*, about the quantity of three eggs or 3½ ozs. of Mosquera beef-meal, and even to get this amount absorbed the bowel must be as far as possible clean and empty. Therefore, after the daily cleansing enema the next injection may with advantage consist of eggs or beef-meal, while in subsequent ones only the whites of eggs should be used. Alcohol is rapidly absorbed, and either ½ oz. of brandy or 2½ ozs. of red wine may be inserted into any enema. Salts and water also are rapidly absorbed.

In arranging the constituents of the enemata for rectal feeding it is important that whatever fluid or solution is employed as a vehicle should be as nearly as possible isotonic with the blood, for if the osmotic pressure should be such as to cause a considerable flow of water into the bowel the absorption of the nutritive constituents of the enema will be greatly interfered with. A 1 per cent. solution of sodium chloride in tap water is isotonic with the blood and is practically essential for the proper absorption of fat and the proteids of raw eggs. Again, it is important that the mucous membrane should be kept clean. The bowel should therefore be washed out once a day with warm 1 per cent. salt solution, and, of course, it is important that the enemata should contain no unabsorbable material. In giving the injections it should be arranged that they shall come in contact with as large an area of mucous membrane as possible, and with this object the hips should be raised on a pillow so that the injection may run up along the colon as far as may be. In arranging the constituents of the enemata one must bear in mind that however well they are absorbed they cannot provide all that the body requires and that the using up of the tissue proteids is the thing above all others to be avoided. In regard to the size of the enema Dr. Beddard says that an adult will, as a rule, easily retain an enema of 9 oz. if properly administered. The following are examples selected from a series of formulæ which are given:—(*a*) Mosquera beef-meal, oz. 3½; salt, gr. 40; water, oz. 9. (*b*) Three eggs; salt, gr. 40; water, oz. 9. (*c*) Somatose, oz. 3½; dextrose (5·8 per cent. solution), oz. 9. (*d*) Arrowroot, oz. 2; raw beef juice, oz. 9.

[1] Guy's Hospital Gazette.

